# Intracardiac echocardiography-guided discrete potential mapping for arrhythmias from right ventricular outflow tract

**DOI:** 10.1093/europace/euaf033

**Published:** 2025-02-15

**Authors:** Nanqing Xiong, Haocheng Ma, Wentao Gu, Jian Li, Weizhuo Liu, Xinping Luo, Henry Hsia

**Affiliations:** Department of Cardiology, Huashan Hospital Fudan University, 12 Wulumuqizhong Road, Shanghai 200040, China; Department of Cardiology, First Affiliated Hospital of Kunming Medical University, Kunming, Yunnan, China; Department of Cardiology, Huashan Hospital Fudan University, 12 Wulumuqizhong Road, Shanghai 200040, China; Department of Cardiology, Huashan Hospital Fudan University, 12 Wulumuqizhong Road, Shanghai 200040, China; Department of Cardiology, Huashan Hospital Fudan University, 12 Wulumuqizhong Road, Shanghai 200040, China; Centre for Cardiopulmonary Translational Medicine, Shanghai Chest Hospital, Shanghai Jiaotong University School of Medicine, 241 Huaihai Xi Road, Shanghai 200030, China; Department of Cardiology, Huashan Hospital Fudan University, 12 Wulumuqizhong Road, Shanghai 200040, China; Division of Electrophysiology, Department of Cardiology, University of California San Francisco, San Francisco, CA, USA

**Keywords:** Right ventricular outflow tract, Ventricular arrhythmia, Ventriculo-arterial junction, Discrete potential, Intracardiac echocardiography

## Introduction

Most idiopathic ventricular arrhythmias (VAs) in the right ventricular outflow tract (RVOT) originate from the ventriculo-arterial junction (VAJ) at pulmonary root.^1,2^ Precise localization of the site of origin (SOO) is required due to limited thickness of the myocardium. Previous studies showed discrepancies in target confirmation and approach for ablation of the arrhythmias.^3^ In this study, we aimed to understand the origin of RVOT-VAs by localizing discrete potentials (DPs) guided by intracardiac echocardiography (ICE).

## Methods

From April 2021 to December 2023, consecutive patients with premature ventricular contractions and ventricular tachycardia originating from the RVOT VAJ area (VA burden > 10%) undergoing ablation guided by ICE and 3D electroanatomical mapping were retrospectively included. The exclusion criteria were confirmed structural or congenital heart diseases, concomitant ablation in left ventricular outflow tract or great cardiac vein, and unsatisfactory ICE image that failed to clearly show RVOT endocardium, pulmonary root, and any pulmonary valve. The study was approved by the local institutional ethics committee.

**Figure 1:**
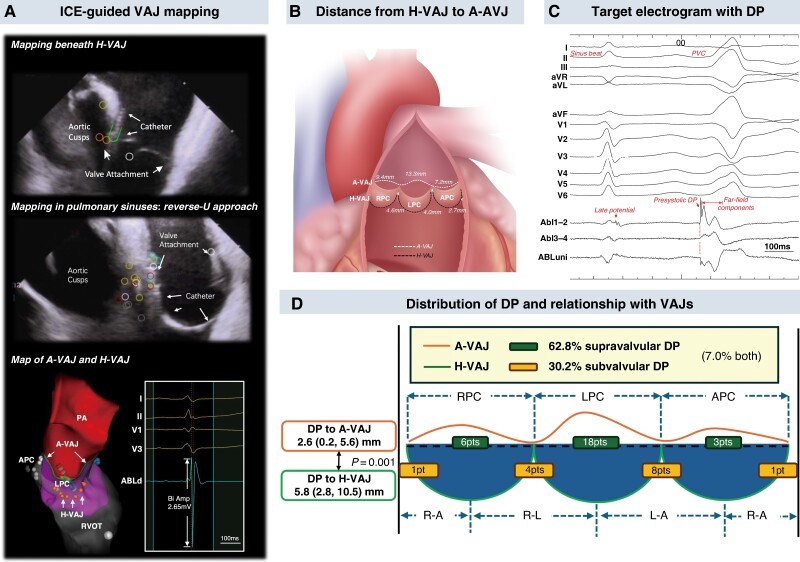
Intracardiac echocardiography-assisted electroanatomical mapping for ventricular arrhythmias from RVOT. (*A*) Catheter position (conventional and reverse-U approach) during ICE-guided VAJ mapping at different locations and the demonstration of H-VAJ and A-VAJ on the 3D map. (*B*) Distance from H-VAJ to A-VAJ at different locations. (*C*) Typical target electrogram with DP in RVOT-PVC. (*D*) Distribution of DPs and their relationship to H-VAJ and A-VAJ. APC, anterior pulmonary cusp; A-VAJ, anatomic ventriculo-arterial junction; DP, discrete potential; H-VAJ, haemodynamic ventriculo-arterial junction; ICE, intracardiac echocardiography; LPC, left pulmonary cusp; PA, pulmonary artery; PVC, premature ventricular contraction; RPC, right pulmonary cusp; RVOT, right ventricular outflow tract.

During procedures, short- and long-axis views of RVOT were acquired with ICE catheter (SOUNDSTAR, Biosense Webster), respectively. Haemodynamic ventriculo-arterial junction (H-VAJ) referred to the level where pulmonary valves were attached, which was superimposed onto the 3D map. Voltage mapping was performed with ablation catheter (Thermocool, Biosense Webster) and a long sheath during sinus beats in six areas, i.e. the middle of three pulmonary sinuses and three sinus junctions. The prolapsed reverse-U catheter position was used to map in all pulmonary sinus cusps (PSCs) (*Figure 1A*). Anatomic ventriculo-arterial junction (A-VAJ) was defined as the level of myocardium demonstrating abrupt decrease of bipolar amplitude. The distance from H-VAJ to A-VAJ was measured in each area (*Figure 1B*).

During activation mapping, DP was defined as^4,5^ (i) sharp high-frequency bipolar potential present as the first deflection of electrogram during VAs, (ii) followed by high-voltage far-field ventricular electrogram with marked difference in slope or an isoelectric segment (*Figure 1C*). The electrogram characteristics at DP sites were analysed.

The initial radiofrequency (RF) was applied at the site of DP, or earliest bipolar electrogram if DP was absent. A successful first RF application was defined when an RF over 30 s resulted in the elimination or an over 90% reduction of VAs compared to the average ventricular beats per minute at baseline, in the next 3 min. Additional lesions around successful sites were created. Univariate and multivariate analysis was performed for independent predictors of a successful first RF application using the logistics regression. Recurrence was defined as the presence of target VAs with burden of >5% at first or sixth month follow-up.^6^

## Results

The data of 56 patients were reviewed. Haemodynamic ventriculo-arterial junction and A-VAJ were identified by ICE-assisted voltage mapping. Anatomic ventriculo-arterial junction was above H-VAJ around the pulmonary root at different distances in three cusps (LPC > RPC *>* APC, all *P* < 0.001). At cusp junctions, the shortest distance from A-VAJ to H-VAJ was found at the junction of APC and RPC (*Figure 1B*).

Acute success was achieved in all patients without major complications. Successful first RF application was observed in 31 (55.4%) patients, which was associated with earlier timing (*P* = 0.013), presence of DP (*P* = 0.008), and higher power setting (*P* = 0.043). After multivariate analysis, it was only associated with the presence of DP (OR 5.11, *P* = 0.038).

Discrete potential was found in 43 (76.8%) patients. Target electrograms with DP had earlier bipolar timing (*P* = 0.022), lower amplitude (*P* = 0.002), and higher number of deflections (*P* < 0.001) compared to those without DP. Anatomically, DPs were found exclusively beyond H-VAJ in 27 (62.8%) patients, in which reverse-U curve was required in 22 cases with DP in the sinuses. In 13 (30.2%) cases, DPs were only recorded below H-VAJ. In 3 (7.0%) cases, DP was observed both above and below H-VAJ. The distance from DP sites to A-VAJ was shorter than to H-VAJ [2.6 (0.2, 5.6) vs. 5.8 (2.8, 10.5) mm, *P* = 0.001] (*Figure 1D*). The DP to H-VAJ distance was correlated with the distance from local A-VAJ to H-VAJ (*P* < 0.001).

Fifty-four patients completed follow-up. Forty-eight (88.9%) had no recurrence at the sixth month. Patients with DPs had a higher success rate (DP: 92.9% vs. no DP: 75.0%, *P* = 0.083). Ventricular arrhythmia burden was significantly lower in DP group at follow-up [first month 0.09% (0.01–0.29%) vs. 0.30% (0.09–10.54%), *P* = 0.030; sixth month 0.08% (0.01–0.37%) vs. 0.24% (0.08–10.10%), *P* = 0.028].

## Discussion

In this study, we used ICE-guided electroanatomical mapping to find that A-VAJ generally extends over H-VAJ around the pulmonary root. Discrete potential was the only independent predictor for single-shot VA elimination, which was closer to A-VAJ than to H-VAJ.

In our approach, an integrated map was created in straightforward cases with H-VAJ defined by echocardiographic imaging and A-VAJ defined by voltage mapping. The value of ICE has been demonstrated in various VAs.^7^ It showed the greatest benefit in our study by defining H-VAJ in advance to guide catheter manipulation during A-VAJ mapping, i.e. conventional or reverse-U technique, and therefore provided help in search of DP. Other advantages of ICE included guiding additional lesion based on the relationship between SOO, and H-VAJ and confirming catheter contact during reverse-U manipulation, etc.

Precise identification of the SOO was important to minimize repetitive RF delivery and recurrence of RVOT-VAs.^2^ Previous literatures, however, showed discrepancy in confirmation of SOO and its supravalvular/subvalvular distribution.^3,8,9^ As DP was the only independent predictor for a successful first RF, it was considered as surrogate for SOO. In our study, DPs were found closer to A-VAJ than H-VAJ, which could be remote from the sinus bottom when A-VAJ was way beyond H-VAJ.^10^ This finding indicated the arrhythmia originates from the distal myocardial extension from the myocardium into the pulmonary trunk rather than at the valve level. Therefore, the key approach of RVOT-VAs was seeking the distal end of ventricular myocardial extensions rather than reaching the pulmonary valve attachment. The distance from DP to ICE-defined H-VAJ was correlated with that from A-VAJ to H-VAJ, indicating the SOO could be way above H-VAJ when the suspected area had long myocardial extension.

The study limitation included the following: (i) retrospective design; (ii) the results were not compared to the approach with 3D electroanatomical mapping but without ICE; and (iii) limited mapping density in the small PSCs.

## Conclusions

Definition of VAJs in RVOT-VAs was feasible with ICE-guided electroanatomical mapping. Discrete potential was observed in most cases, serving as an independent predictor for eliminating VAs with single RF. Discrete potential had close proximity to A-VAJ despite variable distance to H-VAJ, indicating the origin of the arrhythmia was from the distal myocardial extension.

## Data Availability

The data supporting the findings of this study are available upon request to the corresponding authors.
